# Radiation-Induced Growth Retardation and Microstructural and Metabolite Abnormalities in the Hippocampus

**DOI:** 10.1155/2016/3259621

**Published:** 2016-05-08

**Authors:** Shaefali P. Rodgers, Janice A. Zawaski, Iman Sahnoune, J. Leigh Leasure, M. Waleed Gaber

**Affiliations:** ^1^Department of Psychology, University of Houston, Houston, TX 77204, USA; ^2^Hematology-Oncology Section, Department of Pediatrics, Baylor College of Medicine, Houston, TX 77030, USA; ^3^Department of Biology & Biochemistry, University of Houston, Houston, TX 77204, USA

## Abstract

Cranial radiotherapy (CRT) increases survival in pediatric brain-tumor patients but can cause deleterious effects. This study evaluates the acute and long-term impact of CRT delivered during childhood/adolescence on the brain and body using a rodent model. Rats received CRT, either 4 Gy fractions × 5 d (fractionated) or a cumulative dose of 20 Gy (single dose) at 28 d of age. Animals were euthanized 1 d, 5 d, or 3.5 mo after CRT. The 3.5 mo group was imaged prior to euthanasia. At 3.5 mo, we observed significant growth retardation in irradiated animals, versus controls, and the effects of single dose on brain and body weights were more severe than fractionated. Acutely single dose significantly reduced body weight but increased brain weight, whereas fractionation significantly reduced brain but not body weights, versus controls. CRT suppressed cell proliferation in the hippocampal subgranular zone acutely. Fractional anisotropy (FA) in the fimbria was significantly lower in the single dose versus controls. Hippocampal metabolite levels were significantly altered in the single dose animals, reflecting a heightened state of inflammation that was absent in the fractionated. Our findings indicate that despite the differences in severity between the doses they both demonstrated an effect on cell proliferation and growth retardation, important factors in pediatric CRT.

## 1. Introduction

Radiation, as a monotherapy or as a component of a polytherapeutic approach, is used to treat malignant cancers due to its high degree of efficacy. Unfortunately, radiation does not distinguish among the types of cells it destroys, and so healthy tissues in and around the cancerous ones are also compromised [1]. Thus, survival carries with it a risk of deleterious side effects as a result of radiation-induced normal brain tissue injury. This presents a unique challenge with regard to the pediatric population, wherein cranial radiotherapy (CRT) is part of treatment for acute lymphoblastic leukemia (ALL) and solid tumors of the brain, which comprise a significant proportion of malignant cancers [2]. The nonspecific radiation-induced perturbations to the developing brain can have adverse consequences, both acute and long-term, for the brain and body [3–9]. Clinical imaging studies provide evidence at the functional network level that CRT has detectable effects on cerebral integrity [10–12], connectivity [13–15], and volume [11, 16–21]. Numerous studies have found changes in metabolites and/or diffusion tensor parameters in the brain after irradiation in rodents. Using magnetic resonance spectroscopy changes in N-acetylaspartate (NAA), glutamate, choline, lactate, taurine, and myo-inositol have been measured in different brain regions after radiation exposure [22–24]. In addition, diffusion tensor imaging (DTI) parameters have been measured in a variety of brain regions, such as the fimbria, external capsule, and corpus callosum, after brain irradiation [23–26]. In this study, we employ these imaging measurements to investigate the effect of CRT on adolescent rats. CRT also disrupts endocrine function by suppressing growth hormone expression [27–29] resulting in growth retardation [9, 30] and significant alterations in body composition and weight are often observed in survivors of radiation-treated childhood cancers [7, 31, 32]. Although there is a great deal of individual variability within survivors in terms of diagnosis, sex, age at treatment, and type, dose, and regimen of treatment, childhood CRT is recognized as being the biggest risk factor for negative neurocognitive and psychosocial outcomes in adulthood, leading to a poorer quality of life overall [33, 34]. As such, it is imperative to characterize the scope and precise mechanisms of CRT-induced damage in order to determine how best to minimize the cost-benefit ratio of CRT and establish effective interventions to mitigate late-onset neurocognitive deficits that are generally irreversible.

It is widely accepted that the adverse response of the normal tissue in the brain to single dose irradiation occurs early, while its response to fractionated irradiation is late. Differences in the expression levels of inflammatory molecules, between single and fractionated doses, have been reported [35]. However, most of these studies have focused on the early phase of radiation damage and little has been done to elucidate the long-term difference between these two regimens. Animal models of pediatric radiotherapy provide the opportunity to examine radiation effects at the cellular, structural, and circuit levels in the developing brain. One of the most devastating effects of radiation is on cell genesis. Animal studies of single dose radiation effects on neurogenesis show a dramatic decrease [36–38], but the effects of fractionated irradiation of the adolescent brain on hippocampal cell genesis have been less studied. In this work, we investigated the effects of single dose and fractionated CRT in adolescent rats on body weight, brain weight, Ki67 (a marker of cell proliferation), and fractional anisotropy (a measure of functional integrity of fiber tracts) in the fimbria fornix as well as metabolite changes in the hippocampus. The hippocampus and its primary efferent projection (the fimbria fornix) are critical for learning and memory [39, 40]. Indeed, it is thought that the cognitive impairments associated with cancer treatment are due in large part to effects on this structure [41–43]. In this paper, we will present data on proliferation, microstructural, and metabolite changes measured in these two structures.

## 2. Methods

### 2.1. Subjects

Fifty-five male Wistar rats were purchased from Harlan Sprague Dawley Inc. (Indianapolis, IN). Animal care was in accordance with guidelines set by the National Institutes of Health Guide for the Care and Use of Laboratory Animals (2011) and all procedures were approved by the Institutional Animal Care and Use Committee of Baylor College of Medicine (Houston, TX). Rats were 24 days old upon arrival and were housed in pairs in a temperature-controlled vivarium under a 12-hour dark/light cycle (lights off 7:00 am–7:00 pm) with unrestricted access to food and water. Body weights were recorded daily during the course of radiation and then weekly and prior to euthanasia for brain harvest. All irradiated rats developed malocclusions involving their upper incisors. These abnormalities took approximately 6 weeks after radiation to manifest. At this point, their teeth were trimmed under isoflurane anesthesia once every week until termination of the experiment. This was also when their weekly weights were recorded. Sham animals were concurrently anesthetized and weighed to control for exposure to isoflurane. The overgrowth of the incisors prevented irradiated animals from being able to consume the standard, hard, food pellets. Therefore, all rats, including shams, were given fresh, water-softened food pellets in their home cages, every day, throughout the course of the experiment. This was initiated immediately after the first session of CRT to ensure that the drop in body weight during the course of radiation was not due to a CRT-induced inability to chew the standard pellets or fatigue/illness that would prevent only the irradiated animals from accessing the pellets on top of their home cages.

### 2.2. Irradiation Procedure

**Figure 1:**
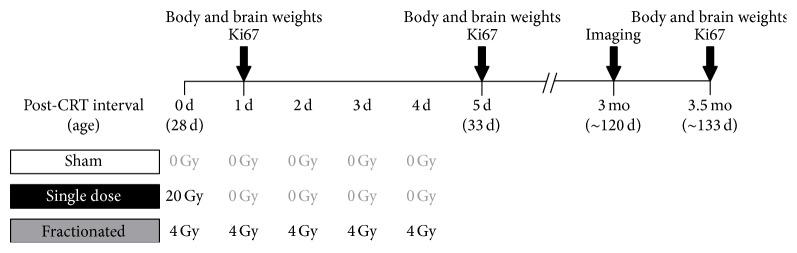
Experiment design and timeline. Note that animals were euthanized at 1 d, 5 d, and 3.5 mo, making this a between-group design.

At 28 days of age, animals were anesthetized using isoflurane and randomly assigned to either the radiotherapy (CRT) or sham group. Animals in the CRT group were individually irradiated at a dose rate of 128 cGy/min using a RS 2000 biological X-ray irradiator 150 kVp, 25 mA (Rad Source Technologies, Inc., Suwanee, GA). Each rat was placed prone and lead shielding was used to ensure that only the region of the head beginning behind the eyes and extending to approximately 5 mm behind the ears received radiation, so that the whole brain (cerebrum and cerebellum), but not the eyes, would be within the field of radiation. Approximately half of the CRT animals received a single dose of 20 Gy (*n* = 19) and the other half received the same dose divided into 4 Gy fractions across 5 consecutive days (*n* = 18). Animals in the sham group (*n* = 18) were anesthetized for the same length of time as those in the CRT group but did not receive radiation. Animals from each group were sacrificed 1 d (*n* = 6 per group), 5 d (*n* = 6 per group), and 3.5 mo (*n* = 6/7 per group) following start of CRT (Figure 1).

### 2.3. Magnetic Resonance Imaging (MRI)

Animals euthanized at the 3.5 mo after CRT interval were first imaged approximately between 3 and 3.5 mo after CRT (Figure 1). A 9.4T Biospec MRI scanner (Bruker, MA) with a 20 cm bore and a quadrature rat brain array (Bruker, MA) were used. All animals underwent spectroscopy followed by DTI. Animals were anesthetized with isoflurane and placed prone on the imaging bed. A respiratory pillow was placed under the abdomen of the animals and a rectal probe was used to monitor respiration and temperature, respectively. The quadrature rat brain array was centered and fixed over the rat brain. Initially, a Tripilot scan was performed to optimize animal placement within the magnet. A T2-rapid acquisition with relaxation enhancement (T2-RARE) scan with the following parameters, TE = 20 ms, TR = 2600 ms, # of averages = 1, 3 cm field of view, 12 slices, 1 mm slice thickness, 1.1 mm interslice distance, and 256 × 256 matrix size, was then performed and used to localize the placement of the spectroscopy voxel. Voxel placement was in the hippocampus (2 mm height, 4 mm width, 3 mm anterior to posterior). Prior to MR spectroscopy a FASTMAP sequence was performed to correct for local field inhomogeneity by adjusting first- and second-order shim coil currents. All MR spectra acquired using a stimulated echo (STEAM) sequence with the following parameters, TR = 2 s, TE = 2.22 ms, # of averages = 512, and voxel size = 24 mm^3^, were executed. Water suppression was achieved using variable pulse power and optimized relaxation delay (VAPOR). For quantification purposes, two scans were obtained, with and without water suppression. MR spectra were then analyzed using LCModel (LCModel, Canada). Metabolites were excluded when they did not pass the criterion of *n* ≥ 4/group and Cramér-Rao lower bounds <20%.

Following spectroscopy, DTI was performed (*n* = 6-7/group) using a spin echo planar imaging (EPI) sequence with a TR = 500 ms, TE = 33.2 ms, 2 repetitions, slice thickness = 12.8 mm, *b* value = 800 s/mm^2^, # of directions 30, and matrix size 128 × 256 × 16 giving a spatial resolution of 150 *μ*m × 150 *μ*m × 800 *μ*m^3^. Total imaging time, for spectroscopy and DTI, was approximately 1 hour per animal. Fractional anisotropy (FA) maps were processed through Medinria and analyzed using Inveon Research Workstation (Siemens, Washington, DC). Briefly, in slices in which the fimbria fornix was visible, a region of interest (ROI) was drawn around the fimbria fornix (left and right sides) and a FA threshold value of ≥0.6 and ≥0.75 applied and mean FA calculated. Mean FA values obtained were averaged between left and fight fimbria and between slices for each animal. The change in fimbria volume caused by radiation was calculated as follows. First, total fimbria volume (TFV) was calculated using a 0.5 FA threshold, to isolate the entire fimbria (Figure 4(a)), in each imaging slice in which the fimbria was visible. Second, a FA threshold value of ≥0.6 and ≥0.75 was applied to the TFV and a resulting thresholded volume (TV_0.6_, TV_0.75_) was calculated (Figure 4(b)). Third, the TV*X* was calculated and averaged between left and fight fimbria and between slices for each animal to arrive at ∑TV_*X*_. Finally, a ratio of ∑TV_*X*_/TFV was calculated (Figure 4(c)).

### 2.4. Brain Tissue Harvest and Processing

Animals were given an overdose of ketamine/xylazine (i.p.) and transcardially perfused with 4% paraformaldehyde at 1 d, 5 d, or 3.5 mo after the start of CRT (Figure 1). Brains were removed, postfixed for 24 h, and stored in 30% sucrose until sectioned. Brains (forebrain and cerebellum only) were weighed and then cut into 50 *μ*m serial, coronal sections on a freezing-stage sliding microtome (Leica Microsystems, SM2000R, Nussloch, Germany) and stored in cryoprotectant in 96-well microtiter plates at −20°C. Every 6th section throughout the rostrocaudal extent of the hippocampus was processed by standard immunohistochemical procedure as reported previously [45] to label proliferating cells. Briefly, sections were rinsed in 0.1 M tris-buffered saline (TBS), quenched for 30 min at room temperature in 0.6% hydrogen peroxide, and rinsed in TBS again. Next, sections were pretreated in 10 mM sodium citrate buffer (pH 8.5) for 30 min in a 80°C water bath, allowed to cool down to room temperature, and rinsed in TBS before incubation in 3% normal donkey serum (Sigma-Aldrich, St. Louis, MO) for 1 h. This was followed by incubation at 4°C for 72 h in primary antibody (rabbit, polyclonal anti-Ki67, 1 : 1800; Vector Laboratories, Burlingame, CA). Sections were then rinsed in TBS, blocked in 3% normal donkey serum for 15 min, and incubated overnight at room temperature in secondary antibody (biotinylated donkey, anti-rabbit, 1 : 250; Jackson ImmunoResearch Laboratories, West Grove, PA). Next, sections were rinsed in TBS, treated for 90 min in avidin-biotin complex (ABC, Vector Laboratories), and rinsed again. Sections were reacted and visualized with diaminobenzidine, rinsed before being mounted onto gelatin-coated slides, and allowed to dry overnight. Sections were then counterstained with methyl green, cleared in xylene, and cover-slipped using Permount mounting medium (Fisher Scientific, Pittsburgh, PA).

### 2.5. Histological Analysis

Ki67+ cells were quantified in the subgranular zone (SGZ) of the dentate gyrus (DG) using a Nikon Eclipse 80i upright microscope in conjunction with StereoInvestigator (MicroBrightField, Williston, VT). Approximately, 10-11 sections per animal were analyzed for Ki67+ cells at 40x magnification throughout the septotemporal axis of the hippocampus by an experimenter blind to the conditions. Since Ki67 staining was sparse, cells were counted nonstereologically, and the number of cells was summed across sections for each animal.

### 2.6. Statistical Analyses

All data were analyzed with Prism 6 (GraphPad Software, Inc., La Jolla, CA). Ki67+ cell counts in the SGZ (1 d, 5 d, and 3.5 mo after CRT), body weights (recorded just before euthanasia at each interval), and normalized brain weights (24 h and 3.5 mo after the end of CRT) were analyzed by two-way ANOVAs with CRT and post-CRT interval as factors. One-sample *t*-tests were used to compare differences between mean brain weight per group (at each interval) and shams (i.e., a value of 100). Mean FA values, fimbria volume-ratios per applied threshold, and hippocampal metabolite levels were analyzed by one-way ANOVAs. Data on body weight, body length, and body weight/length collected from a small, separate cohort of animals (see Figure 6) were analyzed by one-way repeated measures ANOVAs. Bonferroni post hoc comparisons were conducted, where appropriate. Differences between group means were considered significant if *p* < 0.05. Graphs represent mean values or individual data points ±SEM.

## 3. Results

### 3.1. Cell Proliferation

**Figure 2:**
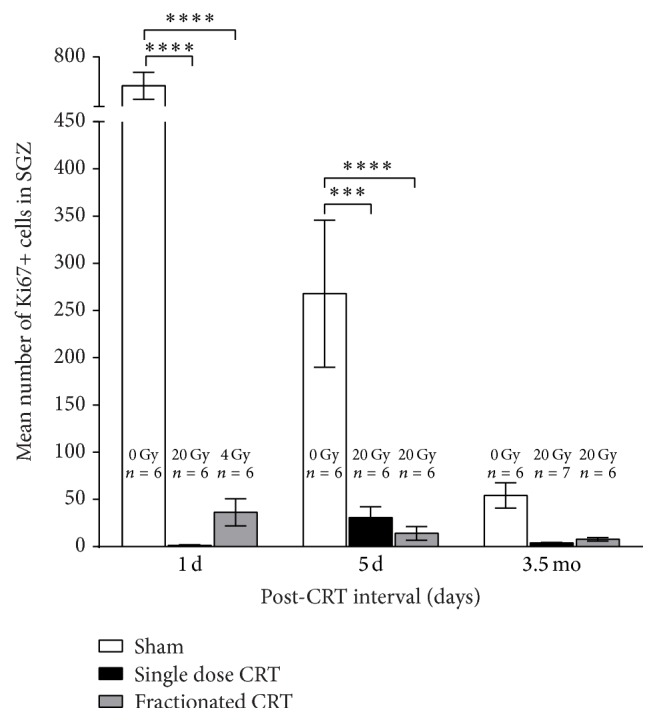
Mean number (±SEM) of Ki67+ cells in the SGZ 1 d, 5 d, and 3.5 mo after CRT [^*∗∗∗*^ *p* < 0.001, ^*∗∗∗∗*^ *p* < 0.0001]. Note that all animals are age-matched at each post-CRT interval and that 24 hours after the start of radiation the single dose CRT and fractionated CRT groups differ only in terms of the cumulative dose of radiation received. The number of Ki67+ cells dropped significantly with age only in the shams.

There was a significant main effect of CRT [*F*(2,46) = 59.72, *p* < 0.0001], post-CRT interval [*F*(2,46) = 20.13, *p* < 0.0001], and interaction [*F*(4,46) = 17.81, *p* < 0.0001] on the mean number of Ki67+ cells in the SGZ of the hippocampus (Figure 2). Post hoc analyses revealed that single dose CRT as well as fractionated CRT significantly reduced the number of Ki67+ cells relative to shams at the 1 d and 5 d post-CRT intervals. However, due to the substantial drop in Ki67+ cells in shams due to age alone [1 d versus 5 d: *p* < 0.0001, 5 d versus 3.5 mo: *p* = 0.001], this difference did not reach statistical significance in adulthood.

### 3.2. Spectroscopy

**Figure 3:**
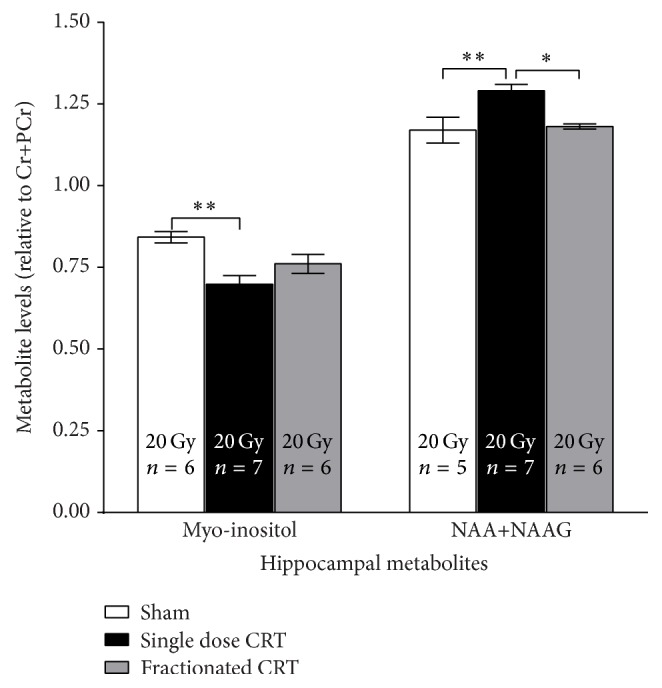
Metabolite changes measured in the hippocampal region at 3 mo after CRT. There was a significant decrease in myo-inositol levels in the single dose group compared to sham, while an increase in NAA + NAAG was measured in the single dose group compared to shams and fractioned CRT [^*∗*^ *p* < 0.05, ^*∗∗*^ *p* < 0.01].

In total, 13 metabolites were successfully measured (*N* ≥ 4/group and ≤20% Cramér-Rao lower bound) in all experimental groups. However, only two metabolites, myo-inositol and N-acetylaspartate + N-acetylaspartylglutamate (NAA + NAAG), had measureable significant change. There was a significant effect of CRT on myo-inositol [*F*(2,16) = 8.3, *p* < 0.01] and NAA + NAAG levels [*F*(2,15) = 8.75, *p* = 0.003] at 3 mo after CRT (Figure 3). Post hoc analyses showed that myo-inositol was significantly lower in single dose CRT animals compared to shams [*p* < 0.01]. NAA + NAAG levels were significantly higher in single dose CRT animals relative to shams [*p* < 0.01] and fractionated CRT animals [*p* < 0.05].

### 3.3. DTI

**Figure 4:**
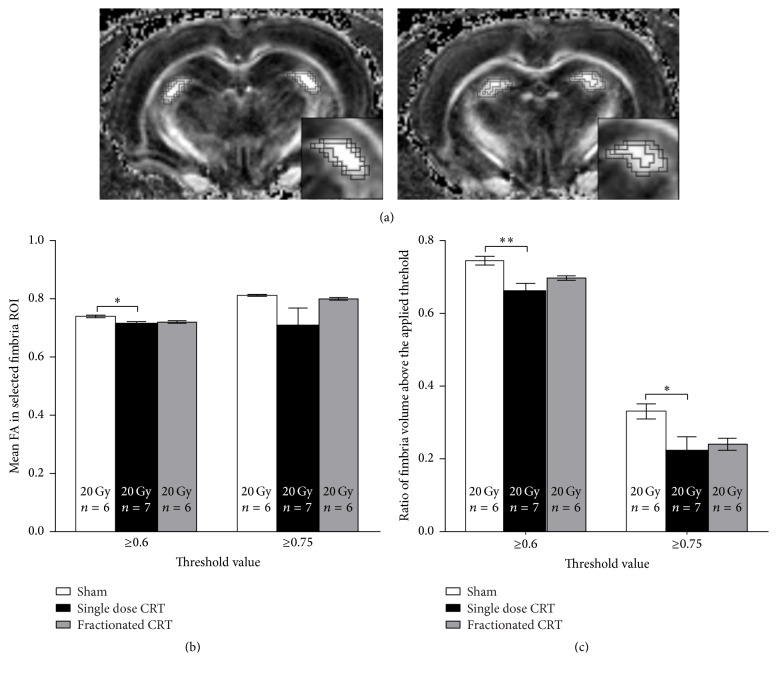
(a) Representational images of the fimbria in sequential cross sections. Concentric thresholded ROIs, representing thresholds of 0.5, 0.6, and 0.75 successively from large to small. Zoomed in insert has been added to better visualize the ROIs. (b) Mean FA values (±SEM) at 3 mo after CRT in the ROI within the fimbria determined by the threshold value. Note that single dose CRT had a reduction in mean FA in voxels ≥0.6 FA. (c) Ratio of fimbria volumes (±SEM) at 3 mo after CRT. There was a reduction in the ratio of the fimbria volume with the applied FA thresholds of ≥0.6 and 0.75 in the single dose CRT animals only [^*∗*^ *p* < 0.05, ^*∗∗*^ *p* < 0.01].

At 3 mo after CRT, no changes in mean FA were found in the fractionated CRT animals. However, single dose CRT animals demonstrated a significant reduction compared to shams [*p* < 0.05] in mean FA values within voxels that were equal to or greater than the set threshold of 0.6 FA (Figure 4(b)). Further, post hoc analyses revealed that, relative to shams, only single dose CRT animals had a significant reduction in the ratio of the fimbria volume above FA thresholds of 0.6 [*p* < 0.01] and 0.75 [*p* < 0.05] (Figure 4(c)). Furthermore, the ratio of the volume of the fimbria above FA thresholds of 0.6 and 0.75 in fractionated CRT was also reduced, albeit not significantly.

### 3.4. Body Weights

**Figure 5:**
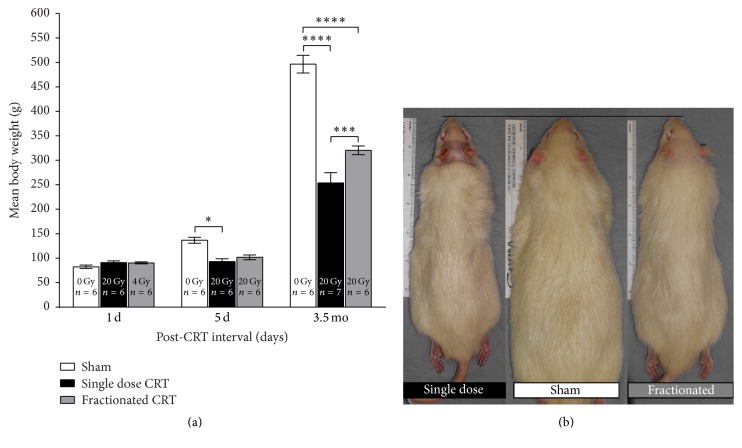
(a) Mean body weights (±SEM) 1 d, 5 d, and 3.5 mo after CRT [^*∗*^ *p* < 0.05, ^*∗∗∗*^ *p* < 0.001, ^*∗∗∗∗*^ *p* < 0.0001]. Note that all animals are age-matched at each post-CRT interval and that 24 hours after the start of radiation the single dose CRT and fractionated CRT groups differ only in terms of the cumulative dose of radiation received. (b) Representative images of a rat from each group 3.5 mo after radiation demonstrate the dramatic retardation of body growth as a consequence of radiation. Note also the permanent effect of single dose radiation on hair growth on the dorsal surface of the region of the head directly in the field of radiation.

**Figure 6:**
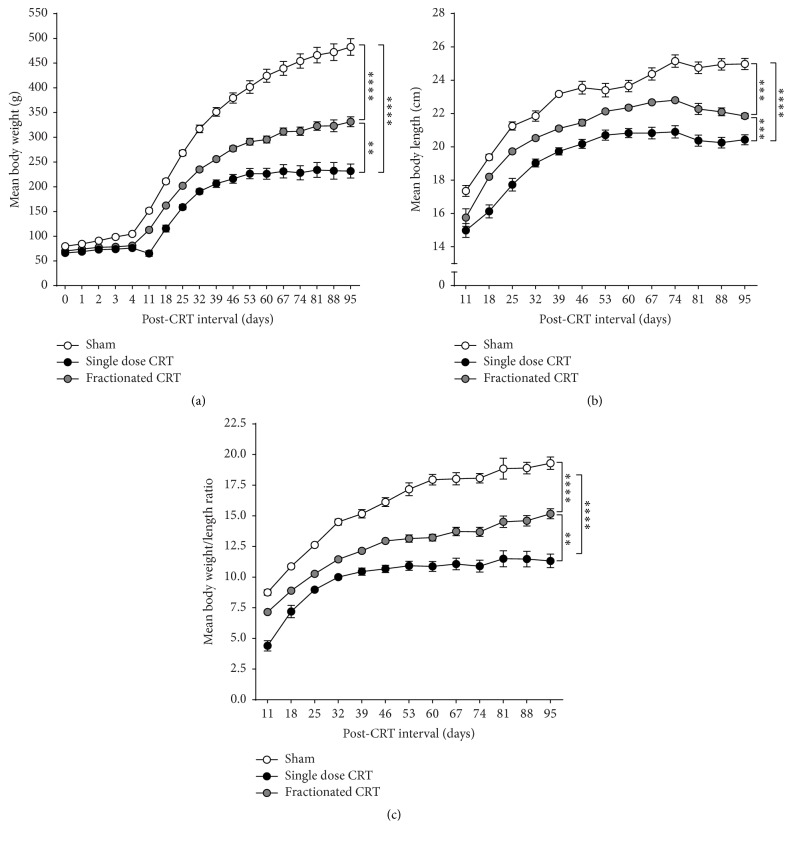
Eight male Wistar rats were irradiated at 28 days of age. They were randomly assigned to single dose CRT (*n* = 4) and fractionated CRT (*n* = 4) and irradiated as described in Methods. Age-matched shams (*n* = 4) were anesthetized but not irradiated. (a) Mean body weights (±SEM) before start of radiation (day 0), during the course of radiation (days 1–4), and every week for 12 weeks after CRT. (b) Mean body length (±SEM) from nose to tail for 12 weeks after CRT. Animals were placed ventral surface down on a surgical drape, while under anesthesia. Tip of the nose and base of the tail were marked on the drape and the distance between them was measured by the same experimenter throughout. (c) Mean body weight/body length ratio (±SEM) [^*∗∗∗∗*^ *p* < 0.0001, ^*∗∗∗*^ *p* < 0.001, ^*∗∗*^ *p* < 0.01].

There was a significant main effect of CRT [*F*(2,46) = 56.55, *p* < 0.001] and post-CRT interval [*F*(2,46) = 556.9, *p* < 0.001] on body weight as well as a significant interaction between CRT and post-CRT interval [*F*(4,46) = 39.14, *p* < 0.001]. Post hoc tests revealed that, at 3.5 mo after CRT, both fractionated and single dose-treated animals had significantly reduced body weights relative to age-matched shams [*p* < 0.0001] (Figure 5). In addition, single dose CRT suppressed body weight even relative to fractionated CRT [*p* < 0.001]. At 5 d after CRT, that is, 24 h after the last fractionated dose of radiation, only the single dose CRT group had significantly reduced body weight relative to shams [*p* < 0.05]. We also measured differences in body length and body weight/length ratios (Figure 6).

### 3.5. Brain Weights

**Figure 7:**
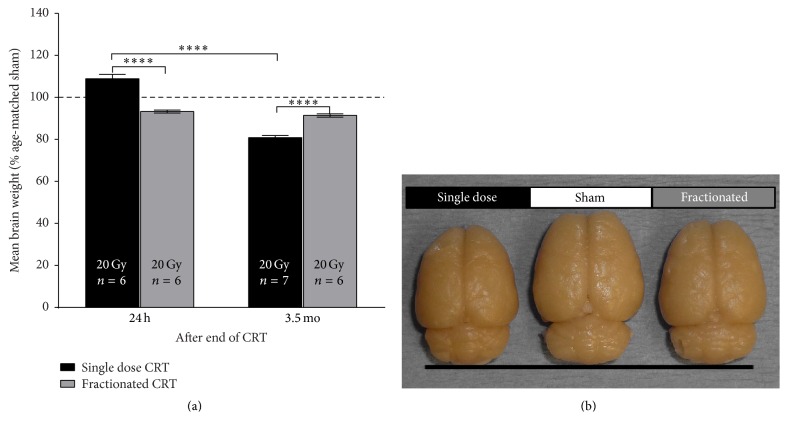
(a) Brain weights expressed as % of age-matched shams (±SEM) 24 h and 3.5 mo after end of CRT. The day after end of treatment, fractionated CRT significantly reduced brain weight by 6.5% compared to shams [*p* < 0.001]. Single dose CRT, however, significantly increased brain weight by 9.2% compared to shams [*p* < 0.01] and 15.7% relative to fractionated CRT [^*∗∗∗∗*^ *p* < 0.0001]. 3.5 mo after end of treatment, fractionated CRT significantly reduced brain weight by 8.4% compared to shams [*p* = 0.0001] and 10.6% relative to single dose CRT [*p* < 0.0001] which reduced brain weight by 18.9% [*p* < 0.0001]. Mean brain weight of the single dose CRT group was reduced 28.1% at 3.5 mo relative to 24 h [*p* < 0.0001]. (b) Representative images of a brain from each group 3.5 mo after radiation highlight the radiation-induced stunting of brain size visible even to the naked eye (figure originally published in [44]).

Brain weights of CRT groups were normalized to that of age-matched shams and compared at 24 h and 3.5 mo following end of treatment (Figure 7(a)). There was a significant effect of interval on brain weight [*F*(1,21) = 141.1, *p* < 0.0001] and a significant interaction between CRT and interval [*F*(1,21) = 108.6, *p* < 0.0001]. Post hoc analyses showed that, the day after end of treatment, single dose CRT significantly increased brain weight relative to shams (9.2% increase, [*p* < 0.01]) and fractionated CRT (15.7% increase, [*p* < 0.0001]). However, in the fractionated CRT group, mean brain weight was significantly lower than sham (6.5% reduction, [*p* = 0.0001]). 3.5 mo after end of treatment, single dose CRT significantly reduced brain weight relative to shams (18.9% reduction, [*p* < 0.0001]) and fractionated CRT (10.6% reduction, [*p* < 0.0001]). The mean brain weight of the fractionated CRT group was also significantly reduced relative to sham (8.4% reduction, [*p* = 0.0001]). Mean brain weight of the single dose CRT group was reduced 28.1% at 3.5 mo relative to 24 h [*p* < 0.0001]. In contrast, there was no change across time in the fractionated CRT group.

## 4. Discussion

### 4.1. CRT Suppresses Hippocampal Cell Proliferation

CRT-induced suppression of cell proliferation in the SGZ was evident the day after the start of radiation and persisted into adulthood (Figure 2). There were no significant differences between single dose and fractionated regimens at any interval, although there were more Ki67+ cells at the 1 d post-CRT interval in the animals that received a single 4 Gy fraction relative to those that received a single 20 Gy dose while this pattern was reversed at 5 d. It may be that there was some recovery in the single dose animals 5 days after their last exposure to radiation or that this is simply an artifact of within-group variance. If assessed independently at each interval, the differences between single dose and fractionated CRT reach statistical significance only at the 1 d interval which is not surprising as at this point the comparison is between doses of 20 Gy and 4 Gy. Note that while proliferation in neither irradiated group differed significantly across intervals, the number of Ki67+ cells in shams dropped significantly as a function of age. Our observations concur with those of others reporting a comparable number of Ki67+ cells in the SGZ of naïve rodents of similar age [38, 46] and a significant drop in neurogenesis between adolescence and adulthood [47–49]. The decrease in proliferation in shams from 1 d to 5 d may in part be a function of error variance and/or due to repeated isoflurane exposure which is reported to suppress hippocampal neurogenesis in juvenile but not in adult rodents [50, 51]. However, studies on isoflurane-induced neurotoxicity typically use long durations of anesthesia, whereas, in our experiment, animals received, on average, 10–15 min (1–3%, 0.5–1 L/min) from days 0–4. Furthermore, as sham animals were matched to irradiated animals for isoflurane exposure, the differences in proliferation between the irradiated groups and age-matched sham groups cannot be attributed to isoflurane. Our results agree with others [38, 52, 53], as independent ANOVAs for each post-CRT interval reveal a significant main effect of CRT even at 3.5 mo after CRT. However, when the data are analyzed as a whole, the significance is lost. We chose a factorial analysis to control for familywise error rate, but the fact remains that, in the absence of data at earlier time points, we would have detected a statistically significant difference at this time point. In summary, our results indicate that 20 Gy of radiation, delivered either as a single dose or in 5 daily fractions, suppresses hippocampal cell proliferation acutely relative to controls, and there is no change in proliferation from the acute to early delayed phase after radiation in both CRT groups.

The SGZ, one of the primary neurogenic niches of the brain, contains progenitor cells which proliferate and subsequently migrate to the adjacent granule cell layer whereupon they differentiate and mature. The survival of these neurons is typically determined 11–16 days after mitosis; therefore, any damage sustained during this period could disrupt normal brain development resulting in adverse long-term effects [54]. Our data show that a dose of radiation as low as 4 Gy is sufficient to suppress cell proliferation to approximately 6% of control levels, 24 hours after exposure. This is consistent with evidence that the majority of apoptosis among progenitor cells in the SGZ occurs within 24 hours after radiation [54] and other reports of a long-lasting decrease in proliferation or neurogenesis in the rodent hippocampus following cranial radiation in early development [55–58]. Disruption of hippocampal neurogenesis during development, especially if persistent, may well contribute to the decrease in brain size that we have observed [59, 60] (Figure 7(b)) as well as late cognitive sequelae of childhood CRT [43, 61, 62].

### 4.2. Microstructural and Metabolite Changes following Single Dose CRT

To assess radiation-induced damage, we used DTI, a noninvasive MRI technique commonly used to measure the diffusion of water within the brain, which can be altered by organizational or microstructural changes in tissue matter [63]. A decrease in FA value (an increase in unrestricted water movement) in the brain has been correlated with edema, inflammation, and a decrease in neuronal myelination [64]. In our model, a significant decrease in mean FA was measured in the fimbria of animals treated with single but not fractionated dose CRT, implying increased radiation-induced damage compared to fractionated CRT. Our findings are consistent with those of others who have reported that FA decreases after single dose radiation exposure in the fimbria [23, 65] and other fiber tracts [24, 26, 65, 66] but is not detectable following fractionated treatment [25]. Histological studies also report white matter damage following a single exposure to radiation [23, 67–69]. We also found that the ratio of fimbria volume was decreased in the single dose-treated animals at both thresholds applied, whereas the fractionated CRT animals showed no reduction at either threshold. Another study using adolescent mice also reported that volumetric differences in the fimbria emerge as early as 1 week after single dose CRT and show limited subsequent recovery [70]. Given that the fimbria fornix comprises a bundle of afferent and efferent myelinated projections to and from the hippocampus, radiation-induced damage to this white matter tract may contribute to late cognitive effects [69].

Using magnetic resonance spectroscopy, we found that, in the single dose, but not in the fractionated, CRT group, myo-inositol decreased significantly, while NAA + NAAG increased significantly relative to shams. These two metabolites are involved in astrocyte metabolism; myo-inositol, which is an osmolyte and is also involved in membrane turnover [71, 72], is more abundant in astrocytes than in neurons, and the production and metabolism of NAA + NAAG are through a cycle that involves neurons, astrocytes, and oligodendrocytes [56, 57]. Our findings agree with those reported by Atwood et al. [73]. Coupling these metabolites findings with our observed reduction in cell proliferation (Figure 2) seems to indicate that in the case of severe radiation-induced injury, as demonstrated by the single dose CRT, the onset of gliosis and demyelination could offer an explanation for these changes. We have previously reported an increase in gliosis following single dose cranial irradiation that persists for months after treatment [74, 75]. Further, we and others have observed demyelination onset at around three months after irradiation [76, 77]. Further experimentation and maybe the use of NMR spectroscopy of brain tissue samples would be needed to confirm our observation.

### 4.3. CRT Stunts Growth

Within the first few days after the start of radiation, body weights of irradiated animals were lower than those of age-matched shams (Figure 5(a)). While both regimens suppressed growth, only the difference between single dose and sham reached statistical significance at the 5 d post-CRT interval, but, by 3.5 mo, all three groups differed significantly from one another. We have observed the retarding effect of CRT on body weight in other strains of rats as well (Long-Evans hooded and Fisher 344, data not shown). We also replicated our findings of CRT-induced overall reduction in body weight in a small, separate cohort of animals (Figure 6(a)). Single dose CRT animals, again, had the highest reduction in body weight. Note that this cohort was followed over time and when data for 5 d after CRT (i.e., day 4 on *x*-axis, Figure 6(a)) are analyzed by one-way ANOVA, shams weigh significantly more than both single dose [*p* < 0.001] and fractionated CRT [*p* < 0.01] animals. Additionally, we documented significant effects of CRT, post-CRT interval, and CRT x post-CRT interval on body length (Figure 6(b)) and the ratio of body weight to body length (Figure 6(c)). All groups differed significantly from each other in terms of body length and body weight/length ratio and single dose CRT had the most severe impact. Furthermore, while growth eventually plateaued with age for all groups, irradiated animals never recovered to sham levels. However, note that while CRT decreased body weight and length, the rate of weight gain and increase in length was similar across groups. Post-irradiation weight loss in rodents has been reported in mice and rats irradiated with different doses and at different ages [25, 78–81]. In order to mitigate the effects of possible damage to the salivary gland, we provided softened food to the animals inside their home cages on a daily basis, starting immediately after the first session of radiation. However, growth remained stunted even 3.5 mo after treatment.

Our brain weight data at 3.5 mo after radiation show that both single dose and fractionated CRT stunted brain growth (Figure 7(a)). Once again, a single dose of CRT suppressed growth to a significantly larger extent (18.9% decrease) than fractionated did (8.4% decrease). Brains of single dose CRT animals weighed, on average, 10.6% less than those of fractionated CRT animals, which is visible to the naked eye (Figure 7(b)). Other studies have also reported radiation-induced suppression of brain growth in rodents [25, 49, 79, 82, 83]. In contrast, 24 h after the end of CRT, that is, when both groups had received a total dose of 20 Gy, brains of single dose-treated animals were, on average, heavier compared to shams and fractionated CRT animals, whereas brains of fractionated CRT animals weighed less compared to shams. The initial increase in brain weight among single dose-treated animals may be a result of acute inflammation-induced edema which is absent in the fractionated CRT group, because they receive radiation over 5 days which allows for bouts of recovery between exposures that are 1/5th the dose.

Collectively, our findings show that whole brain radiation has long-lasting consequences for growth, paralleling the clinical literature [84–86], and that despite some differences, primarily related to inflammation, CRT delivered in childhood/adolescence causes growth stunting of the brain and body and severe suppression of proliferation. The underlying mechanisms, however, remain to be determined. CRT can inhibit growth through its action on the hypothalamus and/pituitary. Indeed, hypopituitarism is frequently observed in both adults [87–91] and children [84, 88, 92–94] treated with cranial radiation even for nonpituitary cancers. Animal studies also show that cranial radiation disrupts endocrine function in a dose-dependent manner [78, 95]. We are currently investigating to what extent our findings can be explained by the interaction of radiation and the endocrine system.

## 5. Conclusions

To our knowledge, this is the first report of the acute and long-term effects of CRT delivered during childhood/adolescence in an animal model which measures proliferation, microstructural metabolites (using translational imaging tools), and phenotype changes. Importantly, the observed early radiation-induced alterations were present 3.5 mo after exposure coinciding with the early delayed phase of radiation injury. Ongoing investigations in our lab are aimed at evaluating the effects of fractionated CRT on cognition and the inhibition of the radiation-induced inflammatory response as a possible mechanism for mitigating the long-term sequelae.
